# Effect of a fever control protocol-based strategy on ventilator-associated pneumonia in severely brain-injured patients

**DOI:** 10.1186/s13054-014-0689-4

**Published:** 2014-12-15

**Authors:** Yoann Launey, Nicolas Nesseler, Audren Le Cousin, Fanny Feuillet, Ronan Garlantezec, Yannick Mallédant, Philippe Seguin

**Affiliations:** CHU de Rennes. Hôpital Pontchaillou. Pôle Anesthésie-SAMU-Urgences-Réanimations. 2, rue Henri Le Guilloux, 35033 Rennes, Cedex, France; Inserm UMR991, équipe 2: “Stress, Defense and Regeneration”. 2, rue Henri Le Guilloux, 35000 Rennes, France; Université Rennes 1, 35033 Rennes, Cedex, France; Plateforme de Biométrie – Cellule de promotion à la recherche clinique, CHU Nantes, EA 4275 SPHERE “Biostatistics, Pharmacoepidemiology & Human Sciences Research”, UFR de Pharmacie, Université de Nantes. 1, rue Gaston Veil, 44000 Nantes, France; Ecole Nationale Hautes Etudes de Santé Publique. 2, Avenue du Professeur Léon Bernard, 35033 Rennes, Cedex, France

## Abstract

**Introduction:**

Fever is associated with a poor outcome in severely brain-injured patients, and its control is one of the therapies used in this condition. But, fever suppression may promote infection, and severely brain-injured patients are frequently exposed to infectious diseases, particularly ventilator-associated pneumonia (VAP). Therefore, we designed a study to explore the role of a fever control protocol in VAP development during neuro-intensive care.

**Methods:**

An observational study was performed on severely brain-injured patients hospitalized in a university ICU. The primary goal was to assess whether fever control was a risk factor for VAP in a prospective cohort in which a fever control protocol was applied and in a historical control group. Moreover, the density of VAP incidence was compared between the two groups. The statistical analysis was based on a competing risk model multivariate analysis.

**Results:**

The study included 189 brain-injured patients (intervention group, n = 98, and historical control group, n = 91). The use of a fever control protocol was an independent risk factor for VAP (hazard ratio 2.73, 95% confidence interval (1.38, 5.38; *P* = 0.005)). There was a significant increase in the incidence of VAP in patients treated with a fever control protocol (26.1 versus 12.5 VAP cases per 1000 days of mechanical ventilation). In cases in which a fever control protocol was applied for >3 days, we observed a higher rate of VAP in comparison with the rate among patients treated for ≤3 days.

**Conclusions:**

Fever control in brain-injured patients was a major risk factor for VAP occurrence, particularly when applied for >3 days.

## Introduction

In severely brain-injured patients, the incidence of fever in the first week of hospitalization approaches 87% [1]. Numerous studies have noted that fever in these patients is associated with a prolonged length of stay in the intensive care unit (ICU) and hospital, severe physical dependency, cognitive impairment and increased mortality [2,3]. Thus, fever control is a common therapy used to treat patients with severe brain injuries [4]. Nevertheless, specific guidelines concerning the optimal delay, length, means and target of temperature control are lacking [4,5].

From another perspective, several experimental and clinical studies that have assessed fever control in other diseases have suggested that there are harmful effects associated with a fever control strategy [6-9]. These adverse effects of fever control, notably shivering, infectious diseases and/or hypotension, are negative factors affecting the prognoses of brain-injured patients [10,11]. In fact, fever, which is typically defined by an elevation of the core temperature above 38.2°C [12,13], may be an adaptive response to injury when an infectious disease is ongoing [14]. Nosocomial infections, particularly ventilator-associated pneumonia (VAP), are frequently observed in severely brain-injured patients, affecting from 8% to more than 70% of patients [15,16]. Therefore, we designed this study to define the role of a fever control protocol in VAP development during neuro-intensive care.

## Materials and methods

This observational study was conducted between January 2008 and December 2009 in the surgical ICU of a university hospital, which received, among others, severely brain-injured patients. The criteria for selection included a severe brain injury (traumatic brain injury (TBI), subarachnoid hemorrhage (SAH), or intracranial hemorrhage), a Glasgow Coma Scale score ≤8 and a need for mechanical ventilation (MV) for 48 hours or longer. Each selected patient underwent chart review. Patients who had aspiration pneumonia at admission or a brain computed tomography (CT) scan without any lesion or who received MV for less than 48 hours were excluded. This study was submitted to the local ethical committee of Rennes University Hospital (CHU Pontchaillou, Rennes, no. 14.45), which stated that no patient consent was needed.

In 2008, Greer *et al.* suggested that fever had a deleterious effect in brain-injured patients and that there was an evidence-based need to confirm the potential benefit of fever control in this population [2]. Consequently, from 1 January 2009 until 31 December 2009, a fever control protocol was applied in cases in which the core body temperature was >38.2°C. Because the fever control protocol was the only therapeutic change, patients admitted from 1 January 2008 to 31 December 31 2008 served as historical controls. In the fever-control group, patients were prospectively and consecutively included, whereas in the historical control group, patients were identified and selected from the list of admissions to our ICU.

The primary endpoint was to assess whether fever control was a risk factor for VAP. The secondary endpoints were the density of incidence and the incidence of VAP, both nosocomial urinary tract infections (UTIs) and catheter-related bloodstream infections (CRBIs), the duration of MV, the length of stay in the ICU, and the mortality rate.

### Patient management

During the two study periods, brain-injured patients were similarly managed according to identical local protocols based on international guidelines [17,18]. The two major objectives were as follows: 1) to infuse neurosedation combining midazolam and opioids; and 2) to maintain cerebral perfusion pressure ≥70 mmHg. All the patients were orotracheally intubated; VAP prevention measures included hand washing with alcohol based antiseptic, a 30° semi-recumbent patient position, MV with positive end-expiratory pressure >5 mmHg, and oral care based on local application of povidone-iodine solution six times daily [15]. Selective digestive decontamination or subglottic drainage was not performed, and ulcer prophylaxis was not systematically administered. The core body temperature was continuously monitored with a rectal thermal probe (Mon-a-Therm™, Covidien, Mansfield, MA, USA).

Fever control intervention was applied in cases in which the body core temperature was >38.2°C [12]. This protocol was established based on previously published studies [4,19,20] and successively included the following: external cooling, an infusion of 500 mL of cooled (4°C) saline serum and enteral administration of acetaminophen. A neuromuscular blocker (cisatracurium) was also used if shivering occurred. The objective was to reach a core body temperature of 36°C to 38.2°C.

### Data collection and definitions

Gender, age, body mass index, the reason for ICU admission, a history of smoking, a positive alcohol test at admission and CT scan-identified lung contusions involving more than one lobe were recorded for each patient at the time of admission to the ICU. The severity of the brain injury was assessed at admission using the Glasgow Coma Scale score and the Simplified Acute Physiological Score II (SAPS II) [21].

We collected the daily minimum (Tmin) and maximum (Tmax) body core temperature and calculated the mean daily temperature as (Tmax + Tmin)/2 during the first eight days of ICU hospitalization. We reported the delay between ICU admission and the initiation of the fever control protocol, the length of application of the fever control protocol and mortality at ICU discharge. Antibiotic prophylaxis, neuromuscular blocker agents, mannitol and thiopental use was reported for all patients. Additionally, acute respiratory distress syndrome (ARDS), defined as previously [22], and microbiological findings for VAP were reported.

VAP was defined as new and persistent pulmonary infiltrates on a chest radiograph occurring after 48 hours of MV, combined with at least two of the following criteria: 1) purulent tracheal secretions and/or body core temperature >38°C and/or leukocytosis >10,000/mm^3^ or leukopenia <3,000/mm^3^; and 2) microbiological confirmation via an endotracheal aspirate quantitative culture growing ≥10^6^ colony forming units (cfu)/mL [23]. The occurrence of VAP was reported and expressed as the density of incidence, defined as the number of VAP cases per 1,000 ventilator-days. We also defined early-onset VAP as VAP that occurred ≤5 days after admission and late-onset VAP as VAP diagnosed >5 days, according the American Thoracic Society criteria [24].

UTIs and CRBIs were defined according to the CDC guidelines [25,26].

### Statistical analysis

The categorical parameters are expressed as number and frequency. The quantitative parameters are expressed as the mean (standard deviation) if there was a normal distribution or the median (interquartile range) otherwise. The categorical parameters were compared using the Chi-square test or, when necessary, Fisher's exact test. The quantitative parameters were compared using the two-tailed *t*-test (when normally distributed) or with the Mann–Whitney *U* test. The temperature value was recorded on the date of the VAP diagnosis. To determine risk factors for VAP occurrence, we used a competing risk model, as proposed by Fine and Gray [27]. VAP before the 28th day of hospitalization in the ICU was an event of interest but could be precluded by death before the 28th day of hospitalization. Patients were therefore observed from the day of hospitalization to the occurrence of VAP before the 28th day of hospitalization in the ICU, to death before the 28th day of hospitalization, or to censoring (hospital discharge while living, without VAP occurrence at day 28 or before). We first compared patient characteristics according to whether the fever control protocol was used to identify potential confounding factors existing before VAP occurrence. A univariate analysis was first performed. Variables with *P*-values <0.20 were then included in the multivariate competing risk model, and a backward selection was applied. The model was adjusted for the risk factors selected according to use of the fever control protocol. By stratifying the duration of fever control, we analyzed the effect of the duration of fever control on VAP occurrence. The adjusted hazard ratios (HRs) and their corresponding 95% confidence intervals (CIs) were calculated, and the cumulative incidence curves for VAP were determined using the Nelson-Aalen non-parametric estimator. By stratifying the fever control duration, we could also describe the impact of the fever control duration on VAP based on the cumulative incidence curve with the Nelson-Aalen non-parametric estimator. A two-sided *P*-value <0.05 was considered statistically significant. The statistical models were built using SAS 9.3 (SAS Institute, Inc., Cary, NC, USA) and R Core Team (2012) software (R: A language and environment for statistical computing. R Foundation for Statistical Computing, Vienna, Austria).

## Results

**Figure 1:**
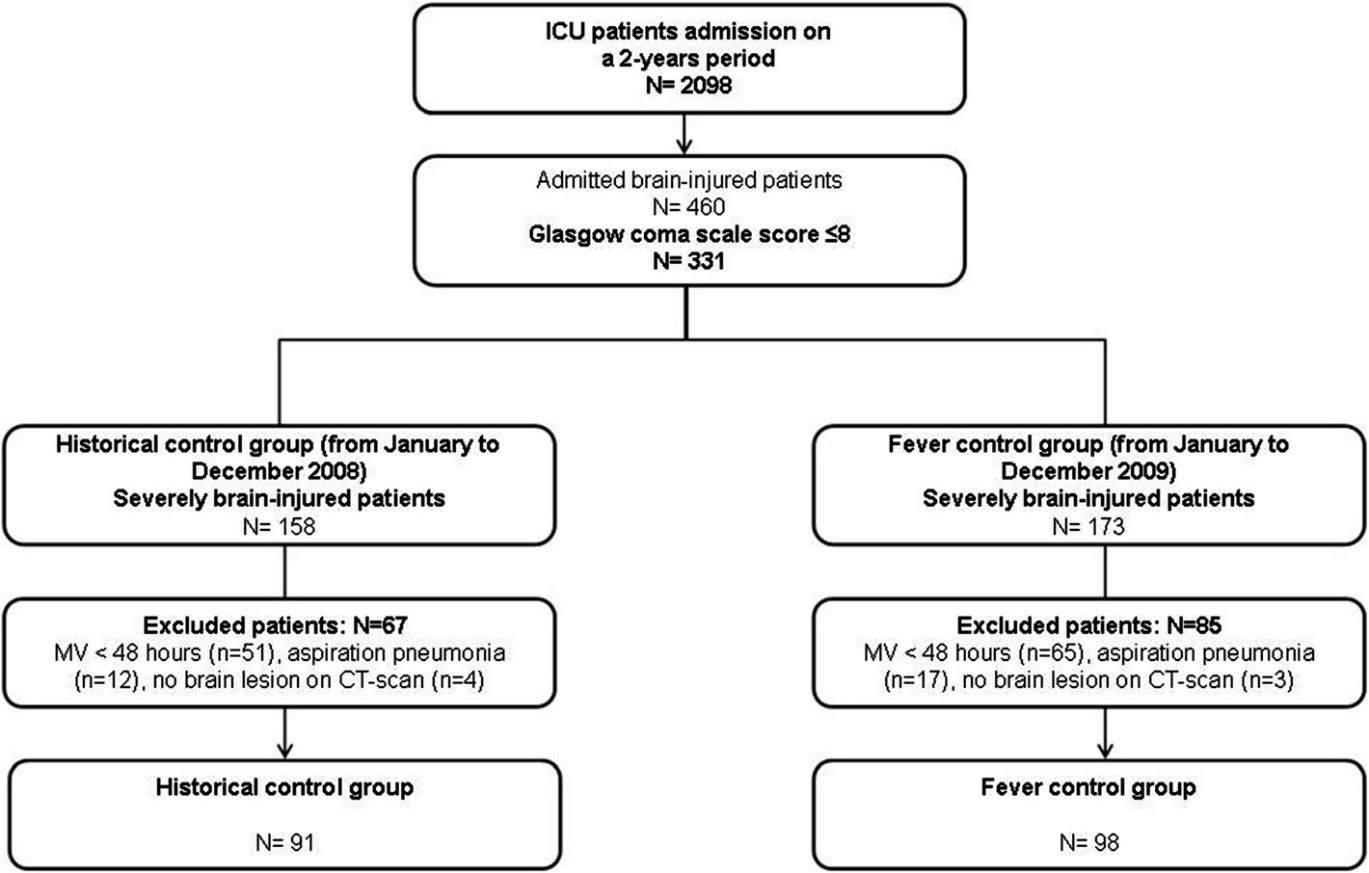
**Flowchart of the study inclusion scheme.** MV: mechanical ventilation.

During the study period, 331 brain-injured patients were screened, and 189 were included (intervention group, n = 98; historical control group, n = 91) (Figure 1). The primary characteristics of the patients are presented in Table 1. The proportion of subarachnoid hemorrhaging was significantly higher in the intervention group than in the historical control group (28% versus 14%, respectively; *P* = 0.006). The clinical characteristics are reported in Table 2.

**Table 1:** **Baseline characteristics**

**Baseline characteristics**	**Intervention group**	**Control group**	***P*** **-value**
	**Number = 98**	**Number = 91**	
**Age**	45 (25 to 57)	53 (27 to 66)	0.022
**Gender (male)**	72 (73%)	64 (70%)	0.631
**BMI**	25 ± 4	24 ± 5	0.123
**Reason for admission**			0.012
**TBI**	64 (65%)	61 (67%)	
**SAH**	27 (28%)	13 (14%)	
**ICH**	7 (7%)	17 (19%)	
**Smoking**	38 (39%)	23 (25%)	0.048
**Positive alcohol test**	11 (11%)	14 (15%)	0.399
**Lung contusion**	28 (29%)	17 (19%)	0.111
**GCS**	6 (3 to 8)	6 (3 to 7)	0.338
**SAPS II**	49 ± 11	53 ± 14	0.013

BMI: body mass index; GCS: Glasgow Coma Scale; ICH: intracranial hemorrhage; SAH: subarachnoid hemorrhage; SAPS II: Simplified Acute Physiology Score II; TBI: traumatic brain injury.

**Table 2:** **Clinical characteristics during ICU management**

**Clinical characteristics**	**Intervention group**	**Control group**	***P*** **-value**
	**Number = 98**	**Number = 91**	
**ARDS, yes**	4 (4%)	2 (2%)	0.684
**Antibiotic prophylaxis**	43 (44%)	43 (47%)	0.645
**NMB agents**	13 (13%)	5 (5%)	0.116
**Pentothal use, yes**	39 (40%)	19 (21%)	0.005
**Mannitol use, yes**	49 (50%)	21 (23%)	<0.001
**ICU length of stay, days**	17 ± 13	11 ± 8	<0.001
**Mortality, number (%)**	23 (23%)	31 (34%)	0.107

ARDS: acute respiratory distress syndrome; ICU: intensive care unit; NMB: neuromuscular blocker.

### Management of the fever control protocol

**Figure 2:**
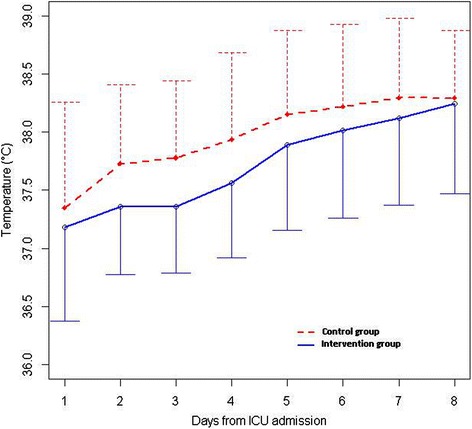
**Mean daily body core temperature curves for the first eight days of ICU stay.** Upper (dashed line) and lower (dark line) limits of standard deviation are displayed for the control group and the intervention group, respectively.

All patients in the intervention group were treated with the fever control protocol when their fever was >38.2°C. The delay before its effective application was less than 24 hours, and its median duration was four days (range, two to six days). The daily mean temperature curves for each group are displayed in Figure 2.

The density of the incidence of VAP was significantly increased in the intervention group beginning on ICU day 5 (26.1 versus 12.5 VAP cases per 1,000 days of ventilation; *P* <0.001). The incidence of VAP was 38% (n = 37) versus 12% (n = 11) for the intervention group and historical control group, respectively. The mean delay for VAP occurrence was similar in the two groups (8 ± 4 days). The mean length of MV was significantly increased in the intervention group (15 ± 11 versus 10 ± 7 days, *P* <0.001). Methicillin-sensitive *Staphylococcus aure*us (MSSA), *Streptococcus pneumoniae* and *Haemophilus influenzae* comprised 80% of the microbiological findings. The microbiological findings are detailed in Table 3. Considering the VAP-onset delay, we reported 10 early-onset VAP cases in the intervention group compared with 5 in the historical control group and 27 late-onset VAP cases in the intervention group compared with 6 in the historical control group.

**Table 3:** **Microbiological findings for VAP**

**Bacterium**	**Total**	**Intervention group**	**Control group**
	**Number = 62**	**Number = 48**	**Number = 14**
**Gram-positive cocci**			
MSSA	22	17	5
MRSA	1	0	1
*Streptococcus pneumoniae*	11	8	3
*Streptococcus anginosus*	1	0	1
**Gram-negative bacilli**			
*Haemophilus influenzae*	17	14	3
*Escherichia coli*	3	3	0
*Moraxella catarrhalis*	1	0	1
*Klebsiella oxytoca*	1	1	0
*Proteus mirabilis*	1	1	0
*Citrobacter freundii*	1	1	0
*Enterobacter cloacae*	1	1	0
*Acinetobacter baumannii*	1	1	0

MSSA: methicillin-sensitive *Staphylococcus aureus*; MRSA: methicillin-resistant *Staphylococcus aureus*; VAP: ventilator-associated pneumonia.

The densities of incidence of UTIs and CRBIs were not significantly different between the intervention and the historical control groups (9.7 versus 14.7 UTIs per 1,000 urinary catheter-days, respectively; *P* = 0.9; 3.9 versus 1.6 CRBIs per 1,000 catheter-days, respectively; *P* = 0.19). The mortality rate also did not differ between the two groups (34% in the intervention group versus 23% in the historical control group; *P* = 0.107).

**Figure 3:**
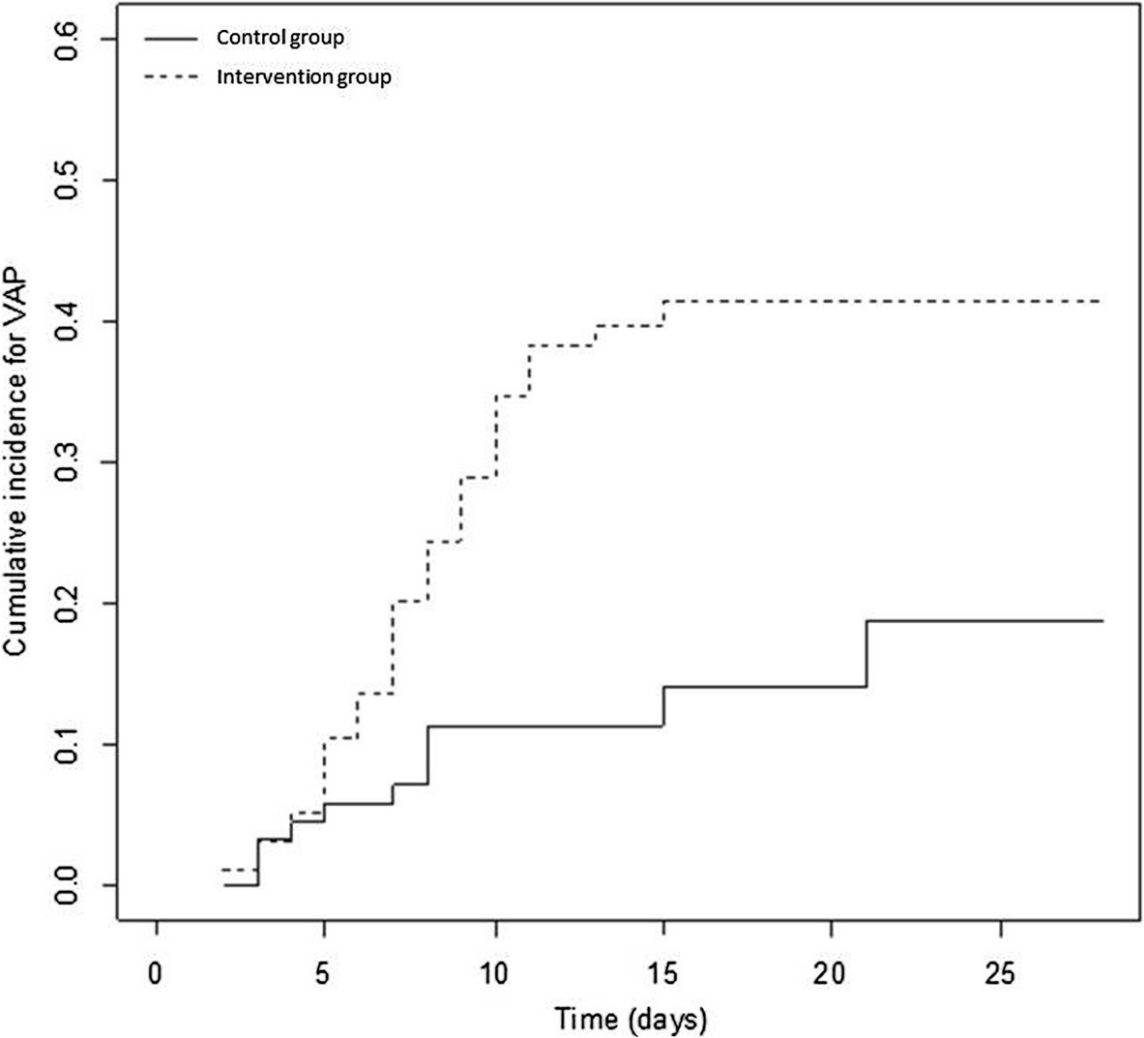
**Cumulative incidence function curve for VAP occurrence according to fever control protocol management.** The non-parametric estimator according to Nelson-Aalen. VAP: ventilator-associated pneumonia.

**Figure 4:**
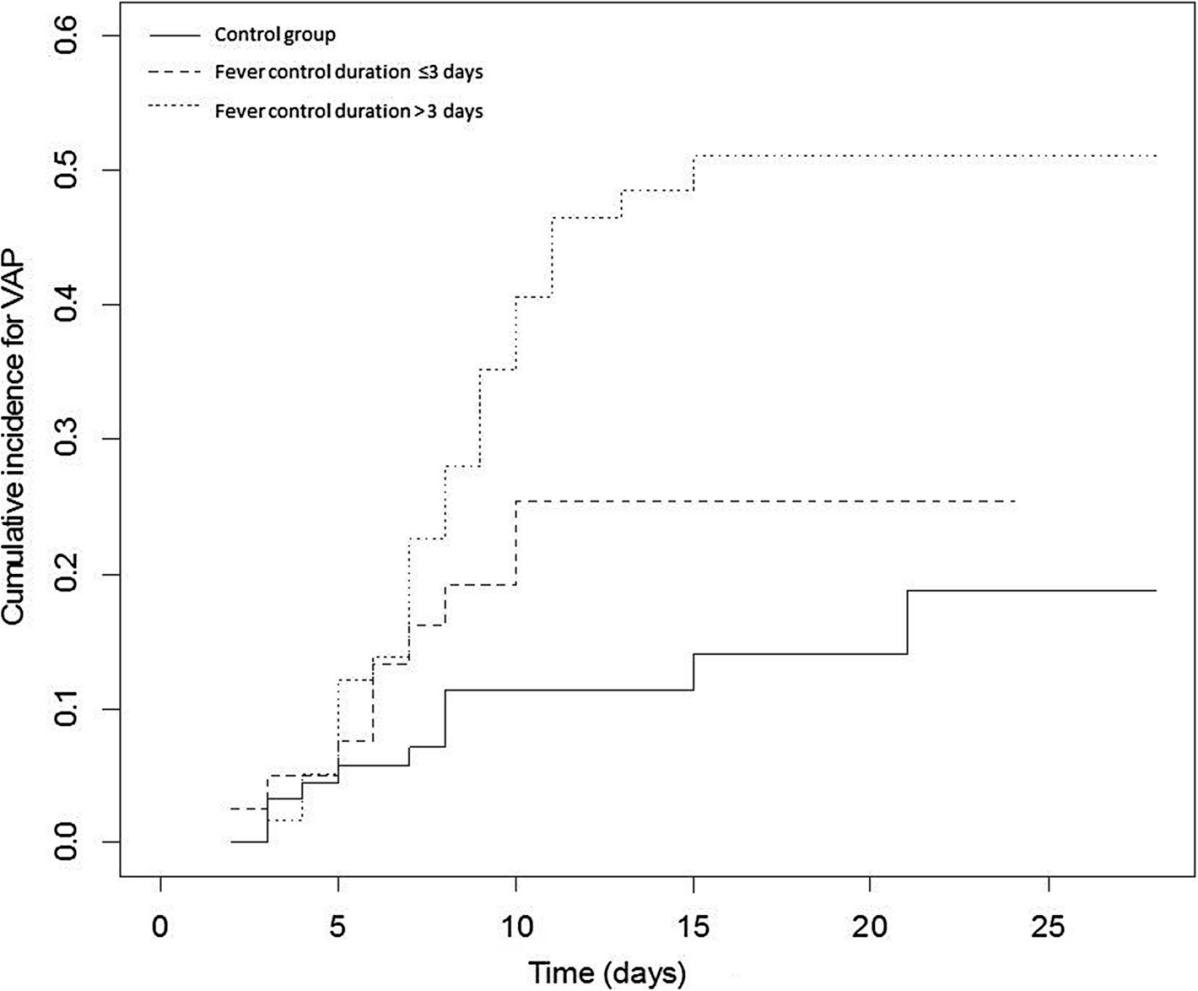
**Cumulative incidence function curve for VAP occurrence according to the duration of the fever control protocol when applied.** VAP: ventilator-associated pneumonia.

In the univariate analysis (Table 4), the patients who developed VAP were significantly younger, used tobacco more frequently and had more extensive lung injury at admission to the ICU. Application of the fever control protocol was a major risk factor for developing VAP (HR 3.06; 95% CI (1.58, 5.94)). In the multivariate analysis, the competing risk model was built after adjustment of the HR for a history of smoking, age, the SAPS II score, pentothal use and the use of neuromuscular blocker agents. The use of the fever control protocol was the only significant independent risk factor for VAP occurrence (HR 2.73, 95% CI (1.38, 5.38)) (Table 4). The cumulative incidence function curves for VAP are displayed in Figure 3. The risk of VAP appeared to be higher when the duration of the fever control protocol was >3 days (Figure 4).

**Table 4:** **Risk factors for VAP occurrence before day 28 (univariate and multivariate analyses)**

**Parameters**	**Univariate analysis**			**Multivariate analysis**		
	**HR**	**95% CI (HR)**	***P*** **-value**	**HR**	**95% CI (HR)**	***P*** **-value**
Age	0.97	(0.95, 0.98)	<0.001	0.97	(0.95, 0.98)	<0.001
Gender (male)	1.82	(0.88, 3.78)	0.110			
BMI	0.99	(0.93, 1.05)	0.730			
Smoking	1.89	(1.09, 3.30)	0.024	1.67	(0.95, 2.93)	0.078
Alcohol intake	0.85	(0.35, 2.09)	0.730			
Lung contusion	2.41	(1.37, 4.24)	0.002			
GCS	0.97	(0.85, 1.12)	0.710			
SAPS II	0.98	(0.95, 1.00)	0.068	1.01	(0.98, 1.04)	0.590
NMB agents	2.11	(0.97, 4.61)	0.061			
Mannitol	1.25	(0.72, 2.17)	0.430			
Thiopental	1.36	(0.78, 2.38)	0.270	0.95	(0.53, 1.69)	0.950
Fever control	3.06	(1.58, 5.94)	<0.001	2.73	(1.38, 5.38)	0.005

BMI: body mass index; GCS: Glasgow Coma Scale; NMB: neuromuscular blocker; SAPS II: Simplified Acute Physiology Score II; VAP: ventilator-associated pneumonia.

## Discussion

In this study, we found that the use of a fever control protocol during the early management of critically brain-injured patients was associated with an increased risk of VAP. During ICU hospitalization of brain-injured patients, sepsis and VAP are major causes of death [23]. In particular, VAP is estimated to occur in 25% to 70% of critically ill patients [23,28,29]. The incidence of VAP in our severely brain-injured patients was 26%, and the microbiological results were relatively similar to those of previous studies [15,30,31], which predominantly found the following microorganisms: MSSA, *Haemophilus influenzae* and *Streptococcus pneumoniae.* It is noteworthy that VAP incidence could be underestimated in the intervention group because fever is a clinical sign of VAP; consequently, the difference in VAP occurrence between the two groups could be minimized. The deleterious effect of such infections has recently been reported in two meta-analyses, which showed that VAP was associated with increased mortality [32,33]. However, in a subgroup of trauma patients, no significant increase in mortality was observed [33].

VAP in severely brain-injured patients has a different pattern than in other critically ill patients. Several factors favor the development of VAP. In brain-injured patients, VAP typically occurs early in the ICU stay, between day 5 and day 11 after hospitalization. The presence of abnormal protection of the glottal area and consecutive early micro-inhalation is typically suggested to explain the occurrence of VAP. These aspirations predominantly contain oropharyngeal bacteria from the commensal flora of the patient, as shown by Ewig *et al.* [34]. In this context, pneumonia results from extensive tracheal and bronchial inocula in patients with altered immune responses and ventilation-related impairment (including due to an endotracheal tube, sedation, or neuromuscular blockers) [35,36].

Fever may be an adaptive response to injury, particularly in cases of ongoing infectious disease. When this response is suppressed, several studies have indicated an increased risk of infectious disease and, especially, VAP. In particular, randomized studies have shown an increased rate of VAP in groups treated with therapeutic hypothermia [37,38]. For instance, although the methodology is debatable, a meta-analysis including eight studies of patients with TBIs reported an increased rate of VAP in the group receiving therapeutic hypothermia (OR 2.37, 95% CI (1.37, 4.10)) [37]. Similarly, Geurts *et al.* found an increased risk of VAP (risk ratio 1.44, 95% CI (1.10, 1.90)) and sepsis (risk ratio 1.80, 95% CI (1.04, 3.10)) in patients treated with therapeutic hypothermia, regardless of what surface or endovascular cooling devices were used [38].

To overcome this adverse effect, normothermia-targeted temperature management has been suggested. However, data on the effects of normothermia in neurologically injured patients are limited. Several studies have investigated and compared the efficacy of different devices in maintaining normothermia but, despite their conclusions, have reported no difference in adverse effects. These studies were not designed to evaluate the rate of infection [39-41]. Additionally, Puccio *et al.* retrospectively compared the effects of a normothermia protocol on TBI management [42]. They found a beneficial effect on intracranial pressure and on the duration of intracranial hypertension episodes in the normothermia group. The systematic initiation of a normothermia protocol, even prophylactically, allowed fever control and possibly attenuation of secondary brain injuries. Similarly, Badjatia *et al*. assessed the effect of normothermia in SAH in a case–control study [11]. They found better fever control, without a significant increase in the rate of VAP (a 58% rate of VAP in the therapeutic normothermia group versus 51% in the historical control group; *P* = 0.08).

In our study, we observed that a sustained intervention for >3 days was more likely to aggravate the incidence of VAP compared with an intervention with a duration of ≤3 days. To the best of our knowledge, no previous study has investigated the effect of the duration of normothermia. It appears that the targeted body temperature does not affect VAP occurrence; however, counteracting the physiological fever response to stress and its duration may be a key factor in the increased occurrence of VAP. More specifically, although fever is deleterious in the short and medium term in brain-injured patients, fever control, whether by hypo- or normothermia, allows reduction of the intracranial pressure at the expense of increased susceptibility to infections. Moreover, in therapeutic hypothermia, the hypothesized anti-inflammatory effect has been associated with impaired cellular and humoral immunity [43]. Unfortunately, to our knowledge, no study has assessed the underlying brain physiology in fever control targeting normothermia.

Concerning the strengths and limitations of our study, the survey of major nosocomial infections in our ICU was prospective starting in 2002, and the diagnostic criteria did not change between the two study periods. Although the use of competing risk models in the multivariate analysis ensured the strength of our study, the major limitation is the design of the study, which used a historical control group, consequently implying limited matching between the two study groups. The interpretation of the results should consider the difference in the mean age and the reason for admission between each group, particularly the higher number of SAH in the control group. Additionally, we have accounted for the confounding factors that may impact the occurrence of VAP, but red blood cell transfusion, level of sedation and antibiotic use during ICU stay were not reported. Head of bed elevation was not reported but standard care in our ICU uses a 30° semi-recumbent patient position. Finally, the fever threshold chosen to start the fever control protocol should be discussed; certain studies have used a temperature threshold of 38°C to start fever control interventions, but solid evidence to clearly support this threshold is lacking.

## Conclusions

Protocol-based control of fever in patients with severe head injuries is associated with an increased risk of VAP during the patients’ ICU stay. Our results suggest that when targeted normothermia is used, the probability of developing an infectious fever within 72 hours is low. However, if fever occurs later, the probability of an infectious fever is higher and should alert intensivists to an ongoing infection. Because data on the impact of fever control and its side effects on the outcome of brain-injured patients are scarce, a large randomized study is required.

## Key messages

The fever control in severely brain-injured patients is a major risk factor for VAP. Moreover, the VAP rate appears to be higher if fever control lasts longer than 3 days.

## Abbreviations

ARDS: acute respiratory distress syndrome
CI: confidence interval
CRBI: catheter-related bloodstream infection
CT: computed tomography
HR: hazard ratio
ICU: intensive care unit
MSSA: methicillin-sensitive *Staphylococcus aureus*
MV: mechanical ventilation
SAH: subarachnoid hemorrhage
SAPS II: Simplified Acute Physiological Acore II
TBI: traumatic brain injury
UTI: urinary tract infection
VAP: ventilator-associated pneumonia
